# THE BRAZILIAN SOCIETY OF BARIATRIC AND METABOLIC SURGERY - SBCBM - PRIORITIZES ENCOURAGING OF SCIENTIFIC PRODUCTION

**DOI:** 10.1590/S0102-6720201500S10001

**Published:** 2015-12

**Authors:** Josemberg Marins CAMPOS

**Affiliations:** President of Brazilian Society of Bariatric and Metabolic Surgery

Worldwide overweight affects approximately two billion people, and Brazil occupies the fifth position with approximately 52% of the population in overweight and obesity. This scenario has contributed to the quantitative increase of bariatric surgery in our country, which is surpassed only by the United States.

In recent years there were also several specialized journals in obesity and bariatric surgery, with an increasing number of publications. Ahmad SS et al^1^., in 2015, reported the 100 most frequently cited articles in bariatric surgery, showing that most of them were originated from the US, followed by Canada and Australia.

Meanwhile, unfortunately, Brazil still has low scientific production; however, it should follow the course of these countries in the search for mechanisms to develop and stimulate interest in research. In addition to other benefits, this could increase the effectiveness and safety of bariatric procedures, benefiting patients and professionals involved, with the consequent growth of the specialty. To achieve this goal, we must seek public and private, national and international partnerships in order to increase the relevance of bariatric research in the country, especially now with the official's performance area in Bariatric Surgery, which will enable the creation of specific training programs.

When preparing the XVII Congress of SBCBM program - aimed at better understanding of the level of scientific activity of Brazilian professionals involved in bariatric surgery -, a review was conducted in Pubmed database, using the headings "bariatric surgery" and "Brazil". In the last three years, were found 655 articles in journals in health field, such as surgery, endocrinology, nutrition, psychology, nursing, dentistry, gastroenterology, physiotherapy, physical education, among others, including international and national publications. Thus, it is clear that we must intensify this target to stimulate and include the largest possible number of Brazilian professionals in scientific atmosphere.

The SBCBM has as major goal encourage learning and dissemination of scientific knowledge. One way for this is to perform basic and advanced courses on topics related to the production of articles, in addition to the creation of the group to support research that seeks greater involvement of all bariatric multidisciplinary team. In recent months, it was carried Scientific Initiation courses and Systematic Review for the training and retraining of our associates.

Following this strategy, in SBCBM Congress 2015 will be an international course on publishing and funding research, which will consider some issues, such as the search for scientific articles on the internet, literature review, writing and science reading, types of studies, video editing, creating and presenting lectures, and other forms of scientific activity, to facilitate access to participants. We also highlight the participation of Dra. Rena Moon, USA, who will present a highly relevant topic for bariatric surgery teams who wish to facilitate the research group training, in order to qualify professionals and improve patient care.

Finally, the Board is pleased to invite the bariatric community to be part of this project, contributing to the scientific growth of our country.

Executive Board of SBCBM - 2015 and 2016Josemberg Campos, Claudio Mottin,
Marcos Leão, Antônio Carlos Valezi, Alexandre Elias, Mauricio Emmanuel, Marcal
Rossi
